# Rotiferan *Hox* genes give new insights into the evolution of metazoan bodyplans

**DOI:** 10.1038/s41467-017-00020-w

**Published:** 2017-04-04

**Authors:** Andreas C. Fröbius, Peter Funch

**Affiliations:** 1 Institut für Allgemeine und Spezielle Zoologie, Abteilung Entwicklungsbiologie, Justus-Liebig-Universität Gießen, Stephanstraße 24, 35390 Gießen, Germany; 2 Department of Bioscience, Aarhus University, Ny Munkegade 116, DK- 8000 Aarhus C, Denmark

## Abstract

The phylum Rotifera consists of minuscule, nonsegmented animals with a unique body plan and an unresolved phylogenetic position. The presence of pharyngeal articulated jaws supports an inclusion in Gnathifera nested in the Spiralia. Comparison of *Hox* genes, involved in animal body plan patterning, can be used to infer phylogenetic relationships. Here, we report the expression of five *Hox* genes during embryogenesis of the rotifer *Brachionus manjavacas* and show how these genes define different functional components of the nervous system and not the usual bilaterian staggered expression along the anteroposterior axis. Sequence analysis revealed that the *lox5*-parapeptide, a key signature in lophotrochozoan and platyhelminthean *Hox6/lox5* genes, is absent and replaced by different signatures in Rotifera and Chaetognatha, and that the *MedPost* gene, until now unique to Chaetognatha, is also present in rotifers. Collectively, our results support an inclusion of chaetognaths in gnathiferans and Gnathifera as sister group to the remaining spiralians.

## Abstract (web summary)

Rotifers are microscopic animals with an unusual, nonsegmented body plan consisting of a head, trunk and foot. Here, Fröbius and Funch investigate the role of *Hox* genes—which are widely used in animal body plan patterning—in rotifer embryogenesis and find non-canonical expression in the nervous system.

## Introduction

Originally discovered in *Drosophila melanogaster* ^1, 2^, *Hox* gene transcription factors have been researched extensively in a phylogenetically diverse range of animals over the last 30 years. Being present in the genomes of nearly all animals with exception of Porifera, Ctenophora, and Placozoa, this family of highly conserved transcriptional regulators controls some of the most fundamental processes of embryonic development. During morphogenesis of all triploblastic metazoans studied, *Hox* genes exhibit expression in body regions along the antero-posterior axis of the embryo at some point. Regional identities are imprinted by either single or combined expression of different *Hox* genes, referred to as “*Hox* code”. This is clearly seen in the segmented taxa Arthropoda^3^, Annelida^4, 5^, and Chordata^6, 7^ where the exact identity of body segments is regulated by the “*Hox* code”. Here, deviations from the appropriate expression profiles result in homeotic transformations of the body regions involved. Amazingly, even across distantly related taxa, body regions comparable to each other (anterior, median, posterior) are patterned by orthologous *Hox* genes. Thus, evolutionary changes of *Hox* gene expression may have led to evolutionary shifts accounting for the emergence of new body plans^8^. Having arisen by tandem duplication, *Hox* genes are often physically linked in genomic clusters. A correlation of the spatial order of *Hox* gene expression along a primary body axis and sometimes even temporal order of *Hox* gene activation during embryogenesis with the order of the genes in the *Hox* cluster, referred to as colinearity^9^, has been observed in a broad range of metazoans. In some bilaterian taxa, however, the *Hox* cluster has been rearranged^10^ or even dispersed up to the relocation of all *Hox* genes to different locations of the genome^11, 12^. Degradation of the *Hox* cluster, however, does not necessarily result in destruction of the *Hox* code. In some cases, specification of appropriate body regions is maintained in taxa where *Hox* genes are no longer closely linked^13, 14^.

Within Protostomia, *Hox* gene expression has been studied in a variety of ecdysozoan and spiralian taxa. However, *Hox* gene activation during embryonic development has not been studied in gnathiferans so far. Based on morphological characters, it has been proposed that the phylum Rotifera including the parasitic Acanthocephala, along with Gnathostomulida and Micrognathozoa, form the clade Gnathifera^15–17^. Rotifers are microscopic, ecologically important aquatic animals comprising *ca*. 2200 described species. Their embryonic development and sexual dimorphic adult body plans exhibit special features. Protected within an egg shell, development starts with a unique cleavage pattern involving an exceptional type of D-quadrant cleavage^18–20^. Lacking a larval form, direct development with early determination gives rise to eutelic animals typically with a tripartite, pseudocoelomic body plan, consisting of a head with a ciliated corona, a trunk and a post-cloacal foot. Several rotiferan tissues are syncytial but musculature and nervous system are cellular^21^. Muscles are mostly formed by few cells directly innervated by nerves formed by simple chains of single neurons^22^. The excretory system consists of paired protonephridia with both cellular and syncytial sections^21^.

Phylogenomic approaches show that rotiferan sequences exhibit very high evolutionary rates resulting in unstable positions within calculated trees^23–25^. Likewise, Platyhelminthes and some ecdysozoans display fast evolving sequences. As a result, Rotifera are prone to long-branch-attraction (LBA) artifacts by grouping with e.g., Platyhelminthes. In addition, taxonomic sampling within Gnathifera is sparse. This has lead to the controversial hypothesis of a clade “Platyzoa” uniting Gnathifera with Platyhelminthes and Gastrotricha within Spiralia (Supplementary Fig. 1)^26, 27^. Recent phylogenomic approaches focusing on resolving spiralian phylogeny, though, support paraphyly of Platyzoa and monophyly of Gnathifera placing Gnathifera basally near the Spiralia/Ecdysozoa split as sister to Lophotrochozoa and Rouphozoa (including Platyhelminthes and Gastrotricha)^28–30^.

Based on the presence of specific amino-acid residues and peptide motifs within the homeodomain and its flanking regions—referred to as signatures—*Hox* genes can be assigned to various paralogous groups (PG1-15)^31, 32^. These paralogous groups are quite clearly defined for anterior class PG1-2, PG3 and median class PG4-5 *Hox* genes. Evolution of these *Hox* genes predates the divergence of Protostomia and Deuterostomia. Due to independendly duplicated median and posterior class *Hox* genes in different bilaterian lineages later on, the exact paralogy status of the remaining median genes (PG6-8) and posterior class genes (PG9-14) is more difficult to determine given the lack of diagnostic position and overall phylogenetic signal. However, these duplication events and subsequent selection present us with *Hox* gene orthologues along with their conserved amino-acid signatures characteristic for these specific lineages in the clades existing today, allowing us to examine phylogenetic relationships based on *Hox* gene complements and the *Hox* signatures within^33^.

In this study, we isolate and examine genes of the *Hox* complement of the monogonont rotifer *Brachionus manjavacas*. Our analysis of *Hox* gene expression during embryogenesis of amictic females shows non-canonical expression patterns in the developing nervous system consistent with an original role of *Hox* genes in neurogenesis. Our sequence analyses show the presence of a new signature in the *Hox6* paralog of *B. manjavacas*, shared by chaetognath *Hox6* genes only. Furthermore, one of the rotifer *Hox* genes possesses median- and posterior-like amino-acid residues, exhibiting similarity to chaetognath *MedPost* genes. These results provide evidence for inclusion of both Rotifera and Chaetognatha in Gnathifera and also support a basal phylogenetic position of Gnathifera as sister group to the remaining Spiralia.

## Results

### Rotiferan *Hox* genes and metazoan phylogeny

**Fig. 1:**
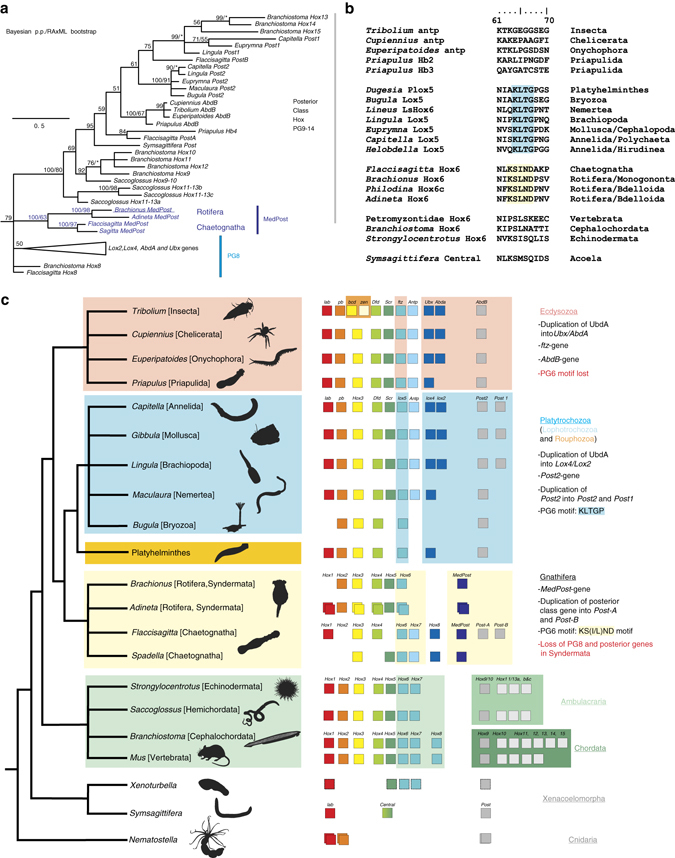
*Hox* gene data places rotifers and chaetognaths in Gnathifera within Spiralia. **a** Phylogenetic tree depicting the relationship of *MedPost* genes to PG8 and posterior class *Hox* genes. Tree topology is from Bayesian analysis. Bayesian posterior probabilities based on 400,000 trees from 40,000,000 generations and ML support values from 1000 iterations are shown above branches. Single values represent Bayesian posterior probabilities only. *Asterisks* denote ML support below 50%. **b** Alignment of ten amino acids of the carboxy flanking region to the homeodomain of PG6 genes. Sequences highlighted with *yellow* contain the new signature found in rotifers and chaetognaths. *Blue highlighting* marks the *lox5*-parapeptide of lophotrochozoan genes. Neither is found in Ecdysozoa, Ambulacraria, Chordata, or Xenacoelomorpha. **c** Summary of representative characteristics of the *Hox* cluster within different metazoan taxa. The tree to the left represents bilaterian phylogeny with Cnidaria as an outgroup. *Boxes* in the middle depict *Hox* gene contingents (color coded according to the assignment of the *Hox* genes to the different paralogous groups) isolated from representative species. The *right hand column* summarizes characteristic *Hox* gene evolution and duplication events along with presence of special *Hox* signatures resulting in *Hox* genes characterizing the respective groups

We isolated single copies of five *Hox* genes from the monogonont rotifer *Brachionus manjavacas*. Based on phylogenetic analyses of the homeodomain and diagnostic amino-acid motifs, we assigned orthology of these genes to the anterior class *Hox* gene PG2 (*Bm-Hox2*), a PG3 gene (*Bm-Hox3*) and central class genes PG4 (*Bm-Hox4*) and PG6 (*Bm-Hox6*) (Supplementary Figs. 2, 3, 4, and 5). The fifth *Hox* gene isolated from *Brachionus manjavacas* surprisingly clusters with *MedPost* genes from the chaetognaths *Flaccisagitta enflata* and *Spadella cephaloptera* (Fig. 1a, Supplementary Figs. 3, 4, and 5) and is strongly supported with a posterior probability of 100% in Bayesian analysis. Maximum-likelyhood (ML) bootstrap support for this grouping is only 63%, this, however, is comparable to the ML support of grouping of the ecdysozoan *AbdB* genes (67%) or all *Saccoglossus kowalevskii Hox11-13* genes analyzed (61%) and even higher than the support for grouping of all *Lox5*-genes undoubtedly related (< 50%) (Supplementary Fig. 5). Mean statistical support from ML analyses for *Hox* gene orthology assignments usually is significantly lower due to the highly conserved nature of the homeodomain^34^. While it could be argued that accelerated evolution could have led to phylogenetic artefacts as LBA, phylogenetic analyses did not reveal branch lengths for the grouping of the chaetognath and rotifer *MedPost* genes significantly larger than those observed for some posterior class *Hox* genes in general.

A careful examination of the homeodomain alignment of *MedPost* genes with either central class *Hox* genes or posterior class *Hox* genes (Supplementary Fig. 3) illustrates both, similarities and differences between rotiferan and chaetognath *MedPost* genes. Both groups share nine of eleven central class diagnostic amino acids: Q (position 6 of the homeodomain), LTR(R/K)RR (26–31) and E (59). Previous work on *MedPost* genes of *Flaccisagitta enflata* ^35^ and *Spadella cephaloptera* ^36^ defined diagnostic posterior class residues characteristic for chaetognath *MedPost* genes: K(3), A(14), R(18), Y(20) and V(21). Only two of these posterior class diagnostic residues, K(3) and Y(20) are shared between chaetognaths and rotifers, but clearly, they represent plesiomorphic characters since they are also present in posterior *Hox* genes of all major bilaterian clades and interestingly also in chaetognath *PostA* and *PostB* genes (Supplementary Fig. 3). The other amino-acid residues A(14), R(18), and V(21) present in chaetognaths but not in rotifers are neither found in posterior class *Hox* genes of Ecdysozoa nor in those of Deuterostomia, but surprisingly in *Post1* genes typical of Lophotrochozoa (Supplementary Fig. 3). Amino-acid residues supposedly characteristic for a new gene class thus have to be re-evaluated once new genes sharing these characteristics have been isolated. *MedPost* genes in general might be defined by only some of the posterior class specific amino-acid positions while others might be specific for one of the taxa only. *Hox* genes belonging to the same paralogous group often exhibit diversity at some positions of the homeodomain (Supplementary Fig. 2). The presence of *MedPost* could also be interpreted as a possible ancestral character, but if it was lost in the Lophotrochozoan + Rouphozoan lineage chaetognaths could still be affiliated to gnathiferans.

The possible close relationship between Rotifera and Chaetognatha was further supported when the homeodomain and 3′ flanking sequences of the PG6 gene *Bm-Hox6* were analyzed (Fig. 1b). Lophotrochozoans and Platyhelminthes possess some central class *Hox* genes containing amino-acid motifs not observed in ecdysozoan or deuterostome taxa. These genes have been named *Lox5* (PG6/7), *Lox2*, and *Lox4* (both PG8). *Lox5* orthologs possess the motif “KLTGP” in the carboxy flanking region of the homeodomain. (position 64–68)^31, 37^, and it seems likely that this motif was present in a common ancestor of Lophotrochozoa and a newly proposed clade Rouphozoa^30^ consisting of Platyhelminthes and Gastrotricha. Importantly, the PG6 gene of *B. manjavacas* not only lacks this “*Lox5*-parapeptide”, it possesses a new *Hox* signature “KS(I/L)ND” at position 63–67 also identified in PG6 genes of the bdelloid rotifers *Philodina roseola* and *Adineta vaga*, and the chaetognath *Flaccisagitta enflata* (Fig. 1b). This motif is not entirely identical among rotifers and chaetognaths, however, *Hox* signatures exhibit some variability as known from the variant *Lox5* signature of Myzostomida^38^ and some Platyhelminthes^39^ or the *Ubd-A* peptide of Spiralia^35^. Recent phylogenomic approaches support Ecdysozoa as sister group to Spiralia, and neither ecdysozoan nor deuterostome PG6 genes possess a *Hox* signature in the carboxy flanking region to the homeodomain. Moreover PG6 *Hox* genes are absent in Xenacoelomorpha^40–42^ sister group to Nephrozoa (Deuterostomia, Ecdysozoa, and Spiralia)^43, 44^. Thus both signatures, the “*Lox5*-parapeptide” and the “KS(I/L)ND” signature could have evolved independently after the split of Ecdysozoa and Spiralia. The alternative hypothesis that the new signature is plesiomorphic has weaker support.

Consistent with their absence in the publically available genome of the bdelloid rotifer *Adineta vaga* ^45^, PG8 genes (*Lox2*/*Lox4*/*Ubx*/*AbdA*) and posterior *Hox* genes (PG9-14) were not recovered from the monogonont rotifer *B. manjavacas* suggesting that these genes are missing in Rotifera (Fig. 1c). Another central class gene (PG5) has recently been recovered from the genome of *Brachionus manjavacas* while the presence of the second anterior class gene (PG1) as identified in *A. vaga* ^45^ could not be confirmed (D. M. Welch, personal communication). Interestingly a single PG8 gene with similarity to ecdysozoan and lophotrochozoan PG8 genes and two posterior *Hox* genes sharing key residues with lophotrochozoans, ecdysozoans, and deuterostomes, have been isolated from the chaetognath *F. enflata* ^35^. Posterior class *Hox* genes are also present in the cnidarian *Nematostella vectensis* ^34^ and have been shown to exhibit extraordinary flexibility leading to possible independent evolution in different lineages^46^. Thus, the most parsimonous explanation is that both PG8 *Hox* genes and the posterior genes (PG9-14) have been lost in rotifers (Fig. 1c).

Some phylogenomic analyses found support for a group called Platyzoa consisting of Gnathifera, Gastrotricha, and Platyhelminthes^23, 24, 47^, but morphological characteristics supporting this group have never been strong. All platyzoans are non-coelomate, ciliated animals with worm-like appearance without specialized respiratory or vascular systems^27^. However, most platyzoans are microscopic and aquatic making diffusion an effective transport mechanism and vascular systems and respiratory organs are unnecessary. Developmental features uniting Platyhelminthes, namely spiral cleavage, resulting cell-lineage and the characteristic Müller’s and Götte’s larvae regarded to be modified trochophores can neither be found in gnathiferan taxa nor in gastrotrichs. Overall morphological synapomorphies supporting Platyzoa are hard to find.

The presence of a *MedPost* gene and the differing signature in the PG6 gene of rotifers and chaetognaths points to a close relationship, which is supported by several shared morphological and developmental traits (Table 1). Gnathifera, which is well supported by phylogenomic studies, is named after the presence of complex chitinous jaws used for feeding, which is found in Rotifera, Micrognathozoa, and Gnathostomulida^15, 48, 49^.

**Table 1:** Morphological, developmental, and special characteristics of the *Hox* cluster of spiralian taxa combined provide an informative basis for the phylogenetic relationship of rotifers and chaetognaths

	Chaetognatha	Rotifera	Micrognathozoa	Gnathostomulida	Gastrotricha	Platyhelminthes	Lophotrochozoa
*Morphological characteristics*							
Tripartite body plan with anus terminal of medial body region	+	+	–No anus	–No anus	−	–No anus	−^a^
Stomatogastric nerve plexi	+	+	−	+	−	−	−
Additional major nerve plexus in the trunk	+	+	−	−	−	−	−
Lateral sensory antennae	+	+	−	−	−	−	−
Trunk exterior cilitated	−	−	+	+	+	+	+
Complex chitinous structures associated with feeding	+	+	+	+	(−)^b^	–(No chitin)	(+)^c^
Protonephridia	−	+	+	+	+	+	+
*Developmental characteristics*							
D-quadrant cleavage	+	+	?	+	+	+	+
Spiral cleavage	−	−	?	+	−	+	+
4d mesentoblast	−	−	?		−	+	+
PGC specification	Preformation	Preformation	?		Epigenesis	Epigenesis/preformation	Mostly epigenesis
Trochophora larvae	−	−^d^	?	?	−	+	+
*Hox cluster characteristics*							
PG6 *Hox* signature KS(I/L)ND	+	+	?	?	?	−	−
*MedPost* class *Hox* gene	+	+	?	?	?	−	−

^a^Phoronids feature a tripartite body plan with terminal anus

^b^Gastrotrichs possess chitinous pharygeal cuticle

^c^The radula of molluscs is chitinous. Being an autapomorphy of this phylum such structures are exceptional among Lophotrochozoa

^d^The body plan of a planktotrophic rotifer resembles a neotenic larva similar to trochophores of Lophotrochozoa; however, there are no separate larval and adult stages

Even though chaetognaths do not possess internal structures quite comparable to the jaws of gnathiferans, both the high chitin content of the spines and teeth and the structure of the chitinous cuticle of the chaetognath head could be homologous to the chitinous parts and membranes of the pharynx in gnathiferans. Both chaetognaths and rotifers feature a tripartite body plan consisting of head, trunk without external motile cilia and foot/tail region. In contrast to Lophotrochozoa and Gastrotricha, the anus is not located terminally but instead near the posterior border of the medial region. Also the trunk regions of Lophotrochozoa and Gastrotricha are with motile cilia. The nervous system includes additional nerve plexi: the caudal ganglion in rotifers and the ventral ganglion in chaetognaths. Both also feature lateral sensory antennae connected to the nervous system. The corona, the ciliary organ of rotifers used for downstream collection of food particles, consists of compound cilia while the corona of chaetognaths is formed by a band of monociliate cells. Despite these structural differences, both are innervated by two coronal nerves. The mastax ganglion of rotifers is connected to the brain via two nerves. In chaetognaths, the suboesophageal ganglion is connected to the brain in a similar fashion with two small separate vestibular ganglia integrated in the nerves connecting to the brain. An additional pharyngeal ganglion has also been reported for Gnathostomulida^50, 51^.

Embryonic development of Chaetognatha and Rotifera shares some important characteristics. Spiral cleavage, prevalent in Lophotrochozoa and Platyhelminthes, is absent in Rotifera and Chaetognatha. The latter two groups exhibit D-quadrant cleavage but do not form a 4d mesentoblast. Primordial germ cells (PGCs) of rotifers and chaetognaths are specified by preformation only, in contrast to the specification of PGCs of Platyhelminthes and Lophotrochozoa by mostly epigenesis^52^. Unfortunately, hardly anything is known about embryonic development of Micrognathozoa and only the earliest embryonic development has been described for a gnathostomulid species once, indicating possible presence of spiralian cleavage in this group^53^. Early cleavage patterns of chaetognaths and rotifers differ from each other. In chaetognaths, cleavage is total and equal forming a blastula. A typical invagination gastrula can be observed. This basic cleavage pattern had originally been mistaken as radial cleavage^54, 55^. Rotifer development involves total and unequal first cleavages. Subsequently columns of cells descending from the A-C quadrant are formed by cleavage with mitotic spindles parallel to the primary axis. The 2D blastomere is then internalized by epibolic gastrulation^19^. These differences do not necessarily contradict the hypothesis of unison of Chaetognatha and Gnathifera. Even within the morphologically and phylogenetically well supported Gnathifera very different cleavage modes can be observed. The development of the parasitic thorny-headed worms Acanthocephala is different from that reported for monogonont rotifers though both are in included in Syndermata^56^. The cleavage modes of rotifers and chaetognaths could, therefore, be interpreted as steps in a transformation series towards spiral cleavage with chaetognaths showing a more basal pattern.

These findings are consistent with the results of phylogenetic analyses based on EST data, mitochondrial genomes and tropomyosin where Chaetognatha is sister group to Lophotrochozoa^57^ or sister group to Protostomia^47, 58^. Moreover, a phylogenomic study based on EST sequences of 197 genes from 66 metazoan species including both Rotifera and Chaetognatha but unfortunately lacking other gnathiferan groups supports this grouping, albeit weakly^25^. Newer phylogenetic studies that took LBA artefacts into account placed Gnathifera as sister to Lophotrochozoa and Rouphozoa but excluded chaetognaths from the analysis. Thus, both Rotifera and Chaetognatha were placed at the same position in different phylogenomic studies, indicating a possible close relationship of these taxa. Intriguingly, the newest phylogenomic study including Gnathostomulida, Rotifera, and Chaetognatha shows strong support for a clade including Gnathifera and Chaetognatha as sister to all lophotrochozoans after Bayesian analysis (posterior probability = 1.0) and mediocre support for a clade formed by Gnathifera and Chaetognatha alone (pp = 0.69)^29^.

### Non-canonical expression of rotifer *Hox* genes

**Fig. 2:**
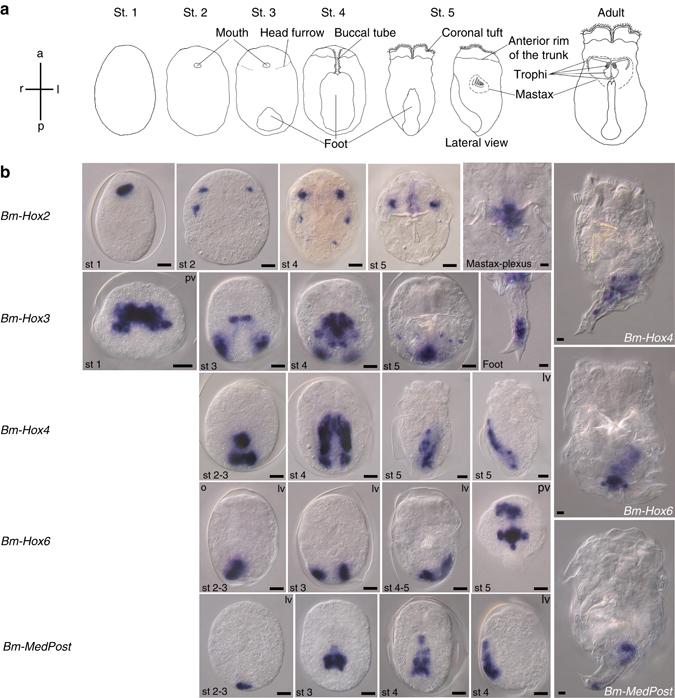
Expression of *Hox* genes during embryogenesis of *Brachionus manjavacas*. **a** Schematic of embryonic stages of *Brachionus manjavacas* with morphological characteristics used for staging. **b** Whole-mount in situ hybridization on amictic female embryos. Adults are only shown for genes with expression persisting into the adult stage. Anterior to the top. Mostly ventral views are shown. pv, posterior view, dorsal side up; lv, lateral view, ventral to the left. Scale bar, 10 µm

**Fig. 3:**
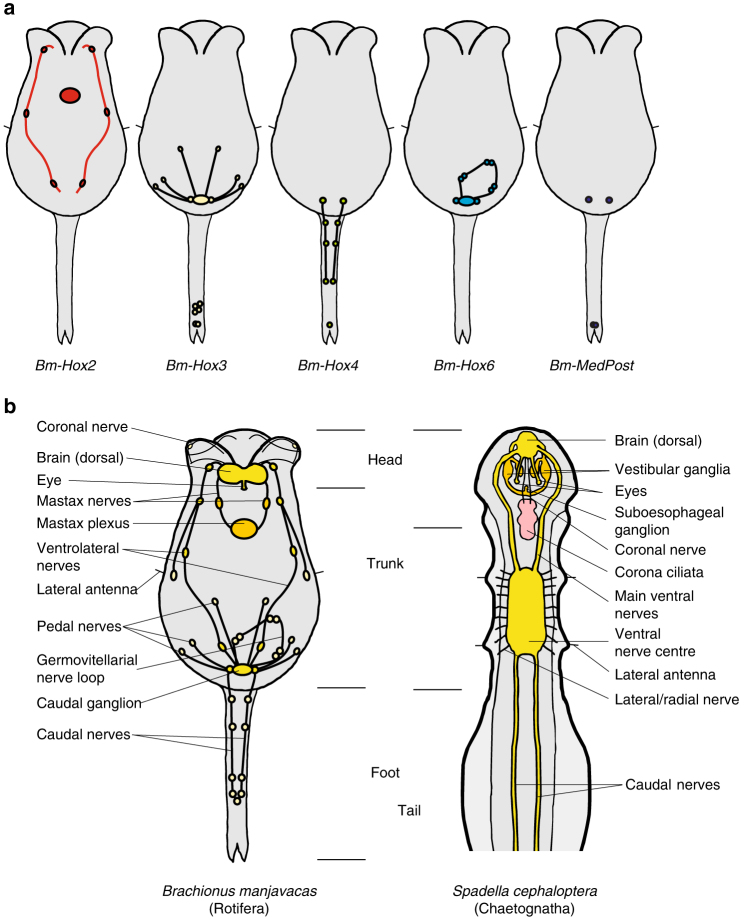
Body plans and nervous systems in Rotifera and Chaetognatha. **a** Diagram of *Hox* gene expression in the nervous system of *Brachionus manjavacas*. **b** Comparison of rotiferan and chaetognath body plans with respect to the structure of the nervous system. Both groups have a dorsal brain and additional nerve plexi: mastax nerves and ganglia in rotifers and vestibular, and esophageal ganglia in chaetognaths as well as a caudal ganglion in rotifers and a ventral nerve centre in chaetognaths with the latter possibly incorporating functional subsets, these are still separated from the caudal ganglion in rotifers, e.g., innervation of sensory lateral antennae

The most fascinating and highly conserved feature of *Hox* genes is the correlation of spatial expression along the anteroposterior axis with the structure of the genomic *Hox* cluster. This spatial collinearity results in the formation of nested *Hox* expression domains by shifting anterior borders of expression in the developing nervous system and other tissues^4^. Unique combinations of *Hox* genes activated within a body region specify that region’s identity (*Hox*-code). Here, we report *Hox* gene expression patterns in Rotifera. During embryogenesis of the monogonont rotifer *Brachionus manjavacas* all five isolated *Hox* genes are expressed in parts of the nervous system and display unique expression patterns unrelated to anteroposterior axis formation (Figs. 2 and 3a).

The anterior class *Hox* gene *Brachionus manjavacas Hox2* (*Bm-Hox2*) is expressed in cells forming the main ventrolateral nerves connecting the brain to the caudal ganglion, a secondary nerve centre at the base of the foot. Initially upregulated in a pair of cells on the ventral side of the embryo near the anterior pole at the beginning of morphogenesis, these *Bm-Hox2*-positive cells move laterally and undergo cell divisions in an anterior-to-posterior fashion resulting in paired nerve cords consisting of three interconnected neurons each (Fig. 2 and Supplementary Fig. 6a). Interestingly, anteroposterior patterning of the main longitudinal nerves by nested expression of several *Hox* genes does not occur. Faint expression of *Bm-Hox2* is also detectable in the mastax plexus after hatching (Fig. 2 and Supplementary Fig. 6d, e), consistent with the observation of anterior *Hox* gene expression in structures associated with the stomatogastric nervous system in other taxa^4^. Expression of *Hox* genes in the rotifer brain or in the coronal nerves (Supplementary Fig. 6b, c) has not been detected.

Strikingly, most *Hox* orthologues (*Bm-Hox3*, *Bm-Hox4*, *Bm-Hox6*, and *Bm-MedPost*) are expressed in a neurogenic region of the embryo near the posterior pole giving rise to morphological innovations: e.g., the caudal ganglion and foot primordium (Fig. 2). The central class gene *Bm-Hox6* is expressed in the developing disc-shaped caudal ganglion originating from a single expression domain shifting inwards from the posterior pole of the embryo. Protruding laterally and ventrally the caudal ganglion resembles a clover leaf. *Bm-Hox3* also participates in patterning of this secondary nerve centre. Bilateral symmetrical domains expressing *Bm-Hox3* at the base of the forming foot fuse during morphogenesis and are integrated into the caudal ganglion. This ganglion serves as the control hub for the posterior part of the trunk providing neuromuscular control of the pedal muscles and innervation of the foot. *Bm-Hox3* is also involved in patterning of the pedal nerves. An expression domain in the distal part of the foot primordium gives rise to six cells with neural morphology in the trunk region connecting to the caudal ganglion. The position of the labeled pericarya is consistent with the six horns of the pedal muscles in *Brachionus*. Remarkably, *Bm-Hox6* was recruited to pattern an additional functional domain of the nervous system, forming an asymmetrical nerve loop on the dorsal left side of the animal connecting the single germovitellarium to the caudal ganglion (Supplementary Fig. 6a). This observation indicates neuronal control of the germovitellarium by or via the caudal ganglion.

The rotiferan foot is a remarkable structure enabling transient attachment to surfaces via a glue-like secretion. Expression of *Bm-Hox4* during foot formation marks cells in the proximal and central parts of the foot, giving rise to the caudal nerves and their connection to the caudal ganglion. Cells clustered at the tip of the foot have long been regarded to be simple gland cells^21^. *Bm-Hox3*, however, is strongly expressed in 12–14 cells connected to the caudal nerves and both *Bm-Hox4* and *Bm-MedPost* exhibit overlapping expression in one or two cells at the base of the “toes”. In deuterostomes, posterior class *Hox* genes play key roles in patterning of the postanal tail^46^. In rotifers, posterior class genes are missing, leaving the key role of modeling of the nervous system of the postanal foot to central and *MedPost* class *Hox* genes. Judging from the innervation of this body region with FMRFamide- and serotonin (5HT)-positive nerve cells (Supplementary Fig. 6f, g) and expression of several *Hox* genes in the foot, we conclude that secretion of the “glue” is under neuronal control and the cells in the foot might represent an additional nerve plexus.

As most rotifer tissues are syncytial, and nerves consist of only a few interconnected neurons^21, 22^, code-like *Hox* expression is somewhat limited. This and newly evolved neuronal structures might have led to *Hox* gene regulation being adapted to modulate functional subsets of the rotifer nervous system (Fig. 3a). That the evolution of new and individual regulatory elements allowed the uncoupling of *Hox* expression from the constraints of collinearity is supported by the dispersed *Hox* cluster structure reported in the bdelloid rotifer *Adineta vaga* ^45^. However, the genomic structure of a monogonont rotifer *Hox* cluster has not yet been published. Surprisingly, *Hox* gene expression in *Brachionus manjavacas* does not seem to violate spatial colinearity completely as the patterns observed exhibit shifting anterior borders of expression to some extent. Temporal colinearity has been reported for some taxa^4^, a correlation of the temporal order of *Hox* gene activation during embryogenesis with the order of *Hox* genes in a cluster. The rather rapid morphogenesis in rotifers complicates determination of the order of *Hox* gene activation in this case. In *B. manjavacas*, transcription of the anterior *Hox* gene*Bm-Hox2* and *Bm-Hox3* is indeed upregulated earlier (stage 1) than transcription of the other *Hox* genes (stage 2). The central class *Hox* genes analyzed are likely to be activated more or less simultaneously, but are all involved in patterning of the caudal ganglion and the nerves of the foot. Differing onsets of transcription may, therefore, be based on the order of morphological processes these genes are involved in rather than correlation with *Hox* cluster structure.

## Discussion

Though not typically used for reconstructing phylogenies, analyses of *Hox* genes for diagnostic residues and conserved motifs give important phylogenetic clues. Patterning of animal body plans during ontogenesis is linked to the *Hox* gene complement in a very unique way. Major evolutionary changes of body plans have been accompanied and most probably have been made possible by changes of the *Hox* cluster structure^6, 8, 59^. *Hox* gene duplications enabled imprinting of additional positional information and thus evolution of additional body regions along the antero-posterior axes of animals. Reflected by possession of conserved amino-acid residues encoded in *Hox* genes these evolutionary changes and gene duplications seem to have happened independently in different clades. *Hox* genes play key roles in axial patterning and segment identity in many taxa. Our findings, however, suggest a different original role of *Hox* genes in metazoan evolution. In the diploblast metazoan *Nematostella vectensis*, *Hox* genes are predominantly expressed asymmetrically on one side of the body column of the polyp exhibiting slightly staggered epithelial expression patterns with large overlap^34^. *Nematostella vectensis Hox* genes are predominantly expressed in the endodermal layer. Neural expression has not been reported. Within triploblast metazoans, recent phylogenomic studies revealed a sister group relationship of Xenacoelomorpha (consisting of Acoela, Nemertodermatida, and *Xenoturbella*) and Nephrozoa (Deuterostomia and Protostomia)^43, 44^. Analyses of *Hox* gene expression in the acoel *Convolutriloba longifissura* indicate possible participation of acoel *Hox* genes in axial patterning of the nervous system due to subepidermal localization and coexpression with neural markers^60^. Consistent with morphological characteristics, phylogenomic analyses place Gnathifera near the base of Spiralia^28–30^. Rotifers possess a miniature but rather complex nervous system. Here *Hox* genes have been recruited to pattern the nervous system in a non-canonical way. Amazingly, rotiferan *Hox* genes show expression domains specifying functional subsets, with a strong bias in patterning of the caudal ganglion and the postanal foot as morphological novelties rather than exhibiting canonical *Hox* code expression along the anteroposterior axis. Unfortunately, *Hox* expression analysis has been reported for only a single chaetognath *Hox* gene^61^; however, expression of *Spadella Hox4* in the ventral ganglion seems to be comparable to *Bm-Hox4* expression in the caudal ganglion. These results might suggest an original role of *Hox* genes in neurogenesis. Consequently, their function in bauplan development would have been co-opted for other tissues during evolution.

Morphological or molecular ambiguities often lead to difficulties in phylogenetic placement of taxa. Several morphological traits strongly support Gnathifera. The support of a grouping of Rotifera and Chaetognatha based solely on either morphological traits (Fig. 3b) or developmental features may be weak; the combined analyses of morphology, development, and *Hox* sequences, however, provide an informative basis for this relationship. In addition, the newest and most comprehensive phylogenomic studies show some albeit moderate support of a close relationship of Gnathifera and Chaetognatha consistent with our results^29^. Though *Hox* gene information from Gnathostomulida and Micrognathozoa is currently not available, we expect these groups to show *Hox* characteristics consistent with this study. Exhibiting ambiguous characteristics indicating a possible close relationship to either Platyhelminthes or Gnathifera, placement of Gastrotricha has always been problematic. Phylogenomic approaches support both, a relationship with Platyhelminthes in Rouphozoa or a close relationship to Rotifera. Based on this study, we suggest an inclusion of chaetognaths in gnathiferans and Gnathifera as sister group to the remaining spiralians. The rather unusual expression of *Hox* genes in *Brachionus manjavacas* is additional evidence of this proposed phylogeny.

## Methods

### Collection of embryos

The rotifer *Brachionus manjavacas* (Florida Aqua Farms) was cultured in 15 ppm artificial sea water (ASW, Tropic Marin Classic) at 24 °C and fed *Nannochloropsis* microalgae culture in ASW (Florida Aqua Farms) twice a day. Animals were collected by sifting through a 50 µm nylon mesh and washed briefly with fresh artificial seawater. After being anesthetized in 0.5 mM Bupivacain in ASW for 12 min, animals and embryos were subjected to prefixation in 0.5, 1, 1.5, 2, 3% formaldehyde in PBS pH 7.4 for 2 min each followed by 3.7% for 10 min at room temperature (RT). For permeabilization of the egg shell, specimens were sonicated in glass test tubes for 40 s. Final fixation took place for 30 min at RT thereafter. Fixative was removed by washing 3–4 times with PTw (1× PBS pH 7.4, 0.1% Tween-20) for 5 min each, tissue was subsequently dehydrated by washing 3–4 times in methanol for 5 min each and stored at –32 °C until use.

### In situ hybridization

Fixed rotifers and embryos were rehydrated briefly in a series of 75, 50, 25% methanol in PTw followed by four washes in PTw for 5 min each. Tissue was permeabilized by treatment with 0.01 mg ml^−1^ proteinase K in PTw for 10 min on a shaker. Digestion was stopped by two 5 min washes with 2 mg ml^−1^ glycine in PTw. After transfer to 1% triethanolamine (TEA) in PTw, specimens were subjected to two treatments with 0.3% acetic anhydride in 1% TEA for 5 min each. After brief washes in PTw the tissue was refixed in 3.7% formaldehyde in PTw for 30 min. Five washes with PTw were followed by a short preincubation in hybridization solution (HYBE: 50% formamide, 5 × SSC pH 4.5, 50 µg ml^−1^ heparin, 0.1% Tween-20, 1% SDS and 100 µg ml^−1^ salmon sperm DNA in diethyl pyrocarbonate (DEPC)-treated water) for 10 min at RT. Prehybridization in fresh hybridization solution was carried out over night at 65 °C. Tissues were hybridized with anti-sense riboprobes (1–3 ng µl^−1^) at 65 °C for 60 h. Subsequently tissues underwent post-hybridization by washing with HYBE twice for 10 and 20 min at 65 °C, followed for 10 min each in 75% HYBE and 25% 2 × SSC, 50% HYBE and 50% 2 × SSC, 25% HYBE and 75% 2 × SSC and 100% 2 × SSC at 65 °C. Two 30 min washes in 0.05 × SSC at 65 °C concluded posthybrization. Tissues were washed for 10 min each in 75% 0.05 × SSC and 25% PTw, 50% 0.05 × SSC and 50% PTw, 25% 0.05 × SSC and 75% PTw and 100% PTw. Blocking was performed by washing five times for 10 min in PBT (1 × PBS, pH 7.4, 0.2 % Triton X-100, 0.1 % bovine serum albumin) and 1 h in 1 × blocking buffer (Roche) in maleic acid buffer (100 mM maleic acid, 150 mM NaCl, pH 7.5) at RT. For detection of the riboprobes tissues were incubated in anti-digoxygenin-AP Fab fragments (Roche) diluted 1:5000 in blocking buffer for 16 h over night at 6 °C on a shaker followed by ten washes for 10 min in PBT at RT. Expression patterns were visualized by three washes in AP buffer (100 mM NaCl, 50 mM MgCl_2_, 100 mM Tris pH 9.5, 0.5% Tween-20) and detection with NBT/BCIP in AP-buffer as substrate. Specimens were analyzed using differential interference contrast optics on an Olympus BX-51 microscope. Digital photomicrographs were captured with a Nikon Coolpix 4500 digital camera (4.0 megapixel).

### Cloning of *Brachionus manjavacas****Hox*** genes

Initially, small fragments of the homeodomain region of *Brachionus manjavacas Hox* genes were amplified by degenerate primer PCR. Different primer sets more or less specific for *Hox* genes in general or *Hox* genes belonging to specific PGs in particular (Supplementary Table 1) were used to isolate *Hox* gene fragments from either mixed stage complementary DNA (cDNA) or genomic DNA (gDNA). In the latter case, presence of introns within the homeodomain was taken into account. gDNA was isolated with an ArchivePure gDNA kit (5prime), cDNA was obtained by RNA isolation (RNeasy kit, Qiagen) followed by reverse transcription (RevertAid First Strand cDNA Synthesis Kit, Thermo Fisher Scientific). Genes were preliminarily identified by BLASTX search (NCBI). Large fragments of cDNAs suitable for phylogenetic analysis and riboprobe synthesis were obtained by RACE (rapid amplification of cDNA ends) with gene specific primers using the SmartRACE Kit (Clontech). All fragments were cloned into pGEM-Teasy vector (Promega) and sequenced at Macrogen Inc (South Korea) or StarSeq (Germany).

### Riboprobe synthesis

Digoxigenin-labeled riboprobes were generated by in vitro transcription using MEGAscript High Yield SP6 or T7 transcription kits (Ambion) with PCR products of suitables clones flanked by SP6- or T7 RNA polymerase promotor sites as templates.

### Orthology assignment and phylogenetic analyses

Assignment to paralog groups (PG) was based on the phylogenetic analyses as well as the presence or absence of diagnostic amino-acid residues or motifs in homeodomain or flanking region of *Hox* genes, commonly regarded as apomorphies for specific PGs and even specific taxonomic groups.

For phylogenetic analyses sequences including the homeodomain and 12 amino acids of the carboxy flanking region next to the homeodomain were aligned using MacVector 8.0. Genome accession numbers of *Adineta vaga Hox* genes and Genbank accession numbers of all other *Hox* gene sequences used in phylogenetic analyses are given in Supplementary Table 2. The most suitable amino-acid substitution model LG + Γ + I was determined by ProtTest 3.4^62^. Bayesian phylogenetic analyses were conducted with MrBayes V3.2.6^63, 64^ on the tera-grid accessible via the CIPRES science gateway V3.3^65^. LG with invgamma was selected with 100% posterior probability with four independent runs of 10,000,000 generations sampled every 100 generations and four chains each. A summary tree was generated from the final 300,000 trees. ML bootstrap analysis was conducted with RAxML-HPC v8.2.9^66^ on XSEDE via the CIPRES science gateway V3.3 with 1000 iterations using the LG + Γ + I model of protein evolution. Final trees were drawn using Figtree 1.4.2 (http://tree.bio.ed.ac.uk/software/figtree/) and CorelDraw 12. Nexus alignments are available upon request.

### Data availability

Additional data associated with this study are available in the Supplementary Information of this publication. Assembled sequences for all *Hox* genes isolated from *Brachionus manjavacas* have been deposited with GenBank under accession numbers KT989538 (*Bm-Hox2*), KT989539 (*Bm-Hox3*), KT989540 (*Bm-Hox4*), KT989541 (*Bm-Hox6*), and KT989542 (*Bm-MedPost*). The amino-acid sequence of *Bm-Hox5* is available in the Figshare Repository under the identifier 10.6084/m9.figshare.4616125.

## Electronic supplementary material

Supplementary Information Supplementary Figures, Supplementary Tables and Supplementary References
