# Supplementary material for: Rotiferan Hox genes give new insights into the evolution of metazoan bodyplans
Source: Nat Commun. 2017 Apr 4;8:9. doi: 10.1038/s41467-017-00020-w (PMC5431905; doi:10.1038/s41467-017-00020-w)
Supplement: Supplementary file 1 — Supplementary Figures, Supplementary Tables and Supplementary References [file 41467_2017_20_MOESM1_ESM.pdf]

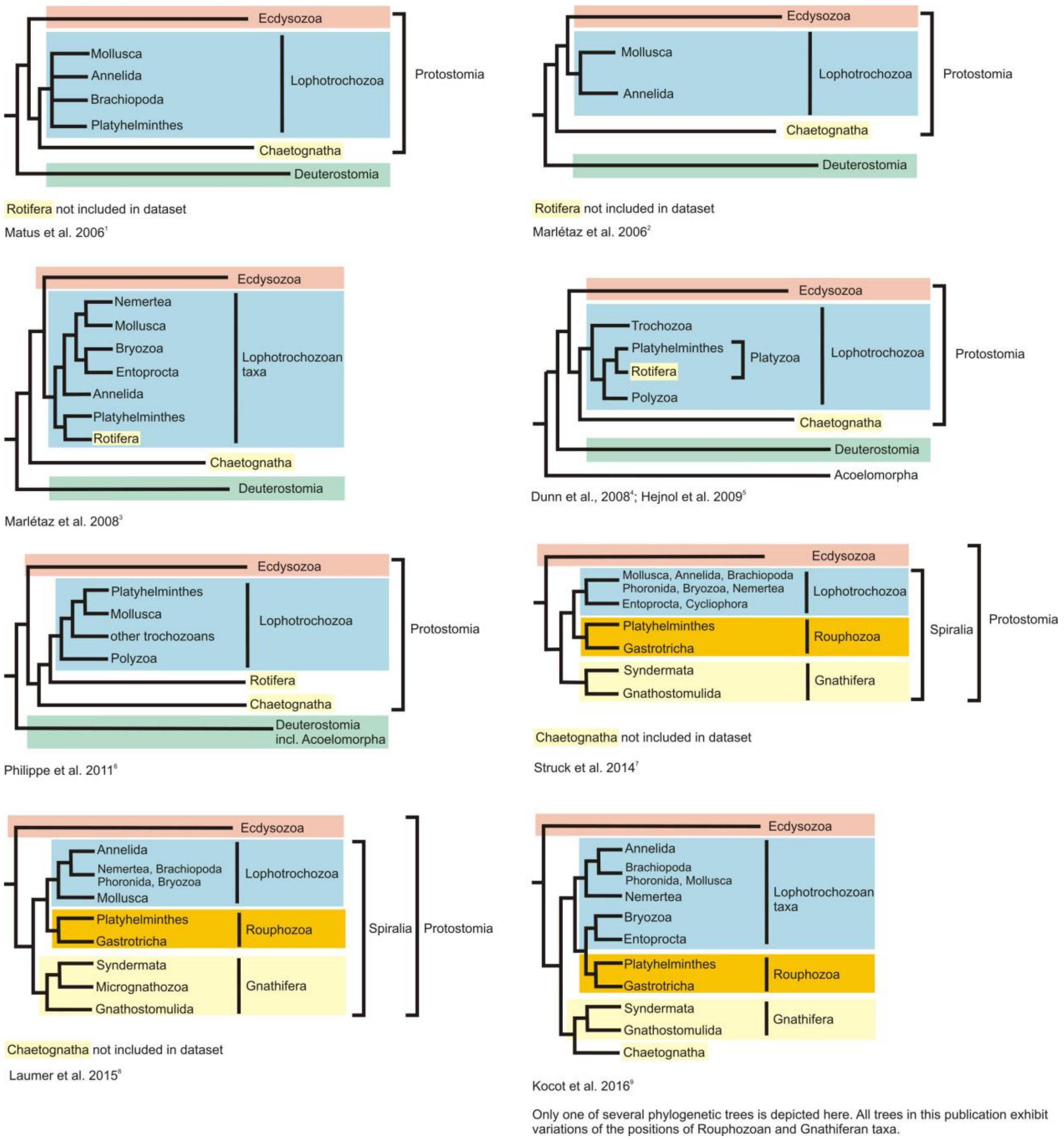

Supplementary Figure 1 | Phylogenetic position of Rotifera and Chaetognatha based on recent large scale phylogenomic studies.

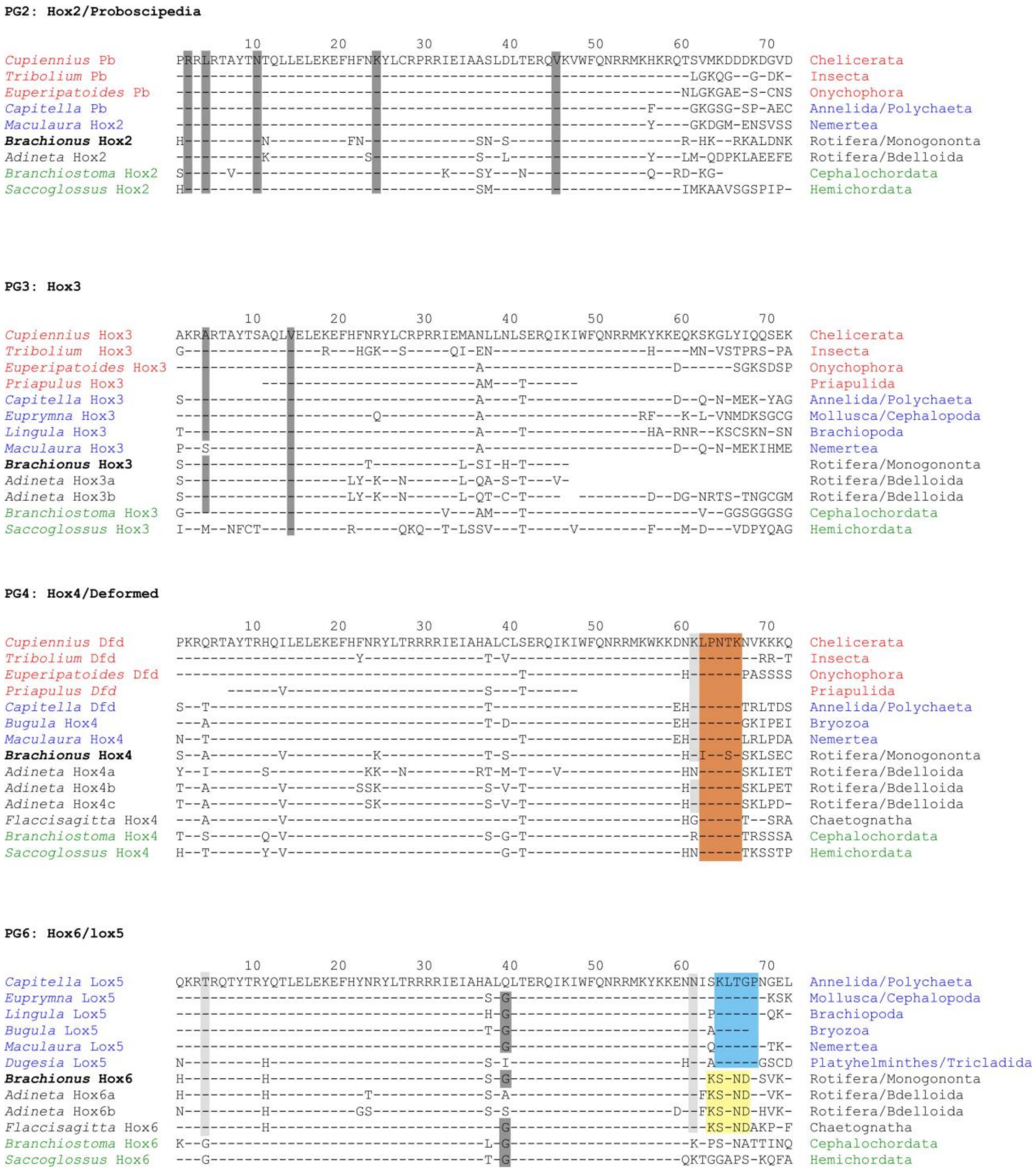

Supplementary Figure 2 | Alignment of *Hox* gene homeodomains and 3' flanking regions of paralog group 2, 3, 4 and 6. Dashes represent identity with respective *Hox* paraloges from *Cupiennius salei* shown in the top line of each group. Ecdysozoan genes listed in red, platytrochozoan genes listed in blue and deuterostome genes listed in green respectively. The following *Hox* signatures are marked: *Hox4*/*Dfd* motif highlighted in orange, *Lox5* parapeptide motif in blue and gnathiferan peptide in yellow. Residues diagnostic for different paralog groups shared by sequences recovered from *Brachionus majavacas* are highlighted in grey: dark grey boxes mark residues found in paralogs across bilaterians and residues boxed in light grey are found in protostomes only.

PG8: Hox8/Lox4/Lox2/AbdA/Ubx

```
                                  10        20        30        40        50        60        70
Capitella Lox4            RRRGRQTYSRYQTLELEKEFQFNHYLTRKRRIEIAHALCLTERQIKIWFQNRRMKLKKERQQIKDLNGLDGK Annelida/Polychaeta
Euprymna Lox4             ----------F----------Y-N---------V----N-S---V--------------K---REM--ACRS Mollusca/Cephalopoda
Maculaura Lox4            ----------------------------R-------S---------------------------E--DSTKD Nemertea
Lingula Lox4              ----------F----------------------V------------------------------EI-DTLK- Brachiopoda
Cupiennius AbdA           --------T-F---------H-------R------------------------------MRAV-EI-EQARM Chelicerata
Tribolium AbdA            --------T-F---------H-------R------------------------------LRAV-EI-EQARR Insecta
Euperipatoides AbdA       --------T-----------H-------R-------V----------------------LRAV-EI-EQARL Onychophora
Cupiennius Ubx            --------T-----------HT------R----M-------------------------I-A--E--EQERQ Chelicerata
Tribolium Ubx             --------T-----------HT------R----M-------------------------I-A--E--EQEKQ Tribolium
Euperipatoides Ubx        -K------T-----------HT------R----M-------------------------M-T-----EQEK- Onychophora
Priapulus Ubx             --------T-----------R-------R----MSQ-----------------------T-AL-EM-AHAKA Priapulida
Capitella Lox2            --------T-----------K--R----R----LS-M------------------E---I-A--E--EKEKT Annelida/Polychaeta
Helobdella Lox2           --------T-----------K--R----R----LS-T-Y----------------E---V-A-RE--EIEKT Annelida/Hirudinea
Urechis Lox2              --------T-----------K--R----R----LS-M----------------                    Annelida/Echiurida
Gibbula Lox2              --------T-F---D-----K--R----R----LS-M------------------E---L-A--E--SPRAP Mollusca/Gastropoda
Brachionus MedPost        K-KH--V---H--F-----YCYSK-------V---GSIK-----V----------E-R-VGKQSH-I-SI-M Rotifera/Monogononta
Adineta MedPost           --KH--V-T-A--F-----YR-SQ-------S---G-VQ-SD--V----------E-R-NLKFNSAVSTSKQ Rotifera/Bdelloida
Flaccisagitta MedPost     H-ER----T-H--A---R-YVT-R----R-----SQS-H-S--------------E-R-KDHVTSPRKNK   Chaetognatha
Spadella MedPost          H-KR------Q--A---R-YVS-R----R-----SQH-D-S--------------ERR-KDHPATTTTSPR- Chaetognatha
Branchiostoma Hox7        -K------T-----------H--K----R---------------------------W---NKLESLKQQPAES Cephalochordata
Branchiostoma Hox8        --------------------H--K----R---------G--------------------AAMLCPPKAETET Cephalochordata
Saccoglossus Hox7         KK-C----T-----------HY-R----R----LS-L-G----------------Y---SKKDDGE-SNQDN Hemichordata
```

PG9-15: Hox9-15/Post1/Post2/AbdB

```
                                  10        20        30        40        50        60        70
Cupiennius AbdB           VRKKRKPYSKFQTLELEKEFLFNAYVSKQKRWELARNLNLTERQVKIWFQNRRMKSKKTSQRNAENNQNNTN Chelicerata
Tribolium AbdB            -------------------------------------------------------N--N---Q-AQQ---N- Insecta
Euperipatoides AbdB       -------------------------------------------------------N--NN---L---K     Onychophora
Priapulus AbdB            --------T-Y-------------------------T---------------------SN-KET-KQRQHQS Priapulida
Priapulus HB4             --S-------Q-IVD----YKV-P-ITR-R-F-M-H--G-S--------------T--HLLTLRV        Priapulida
Capitella Post1           PK--------P-VSA--N-YSTST-IT-AR-K-V--E-D-----I---Y----I-E--IATKR-KVQSDLFM Annelida/Polychaeta
Euprymna Post1            L--R-R----Y-IA---R-YALST-I--SR----SQL---S---I--------I-A--LQK-DETLKTQTP- Mollusca/Cephalopoda
Lingula Post1             M---------Y-IA---R-YVS-T-I--P-----SQR-Q-S--------------E--VKGGKQT        Brachiopoda
Capitella Post2           Q-------TRY--MV--N--IN-S-ITR-----ISCK-H-S-----V--------R--LNE-AKSLIKDHEP Annelida/Polychaeta
Euprymna Post2            G-------TRY--MV--N---NSS-ITR-----ISCK-Q-------V--------R--LNE-AKARLREDRE Mollusca/Cephalopoda
Lingula Post2                 ----TRY--MV--N---N---ITR-----ISCK-H-S-----V--------R--LNE-AKALFKSEDI Brachiopoda
Bugula Post2              S-------TRY--MV--T--IN-S-ITR-----ISCR-R-------V--------R--LND-AKNAQLTVQQ Bryozoa
Maculaura Post2           T-------TRY--MV--N--MT-S-ITR-----ISCK-H-------V--------R--LNS-AKVKSEVENT Nemertea
Brachionus MedPost        K--H-QV--RH--F-----YCYSK-LTRKR-V-I-GSIK----------------E-REVGKQSHLIGSIGM Rotifera/Monogononta
Adineta MedPost           R--H-QV-TRA--F-----YR-SQ-LTRKR-S-I-GAVQ-SD-------------E-RENLKFNSAVSTSKQ Rotifera/Bdelloida
Flaccisagitta MedPost     H-ER-QT-TRH--A---R-YVT-R-LTRRR-I-ISQS-H-S---I----------E-REKDHVTSPRK-K   Chaetognatha
Spadella MedPost          H--R-QT--RQ--A---R-YVS-R-LTRRR-I-ISQH-D-S---I----------ERREKDHP-TTTTSPRK Chaetognatha
Symsagittifera Post       S---R--P-N-------------T-ITRER-L-I--S----D-------------N--QHSGGPGMGPPIPG Acoela
Branchiostoma Hox9        S---C--TR------------Y-M-LTRER-Y-ISQHV--S--------------M--M-KQRQE---P-PP Cephalochordata
Branchiostoma Hox10       G---C--T-Y-I-----------M---RER-Q-IS-HV--SD-------------M-RMNKAREE-IR-HQE Cephalochordata
Branchiostoma Hox11       T---C--T-Y-------------MF-TRER-Q-I--Q----D-------------M-RMK--AMQ-LMEEKQ Cephalochordata
Branchiostoma Hox12       S---C----V-L---------Y-M-ITREQ-G-I--KV---D-------------M-RMK--HEEEAFRAHT Cephalochordata
Branchiostoma Hox13       G---C----Y-LSV--Q-YIQ-R---RET-L--SQR----D--------------Q-RLEF-SGN-T      Cephalochordata
Branchiostoma Hox14       --P--R----Y-LN---N-YVQ-Q-I-RD--LQ-SQK----------------I-Q--LDR-NSEMCP     Cephalochordata
Branchiostoma Hox15       T---R----P-LAL-D-YASQKFLT-E-K-ISESSS-S-M---------------E--LAR-A---RHAQHL Cephalochordata
Saccoglossus Hox9/10      G---C--T-------------M-LTRER-VDI--L--------------------L--QN----TMLH     Hemichordata
Saccoglossus Hox1113a     N------T-Y-----------Y-M-LTRDR-TDI--A---S---I----------L--MRL-EENERKQQDG Hemichordata
Saccoglossus Hox1113b     R-T-----T-M-IF---QA-QN-M-LTRER-TK-SQQ-S-S---I----------L--MTE-EKLEEKELRE Hemichordata
Saccoglossus Hox1113c     R-T--R----L-IF------QQ-M-LTRDR-SR-SQA-------I----------L--MTD-ERNEQDMMQQ Hemichordata
```

Summary

```
                                  10        20        30        40        50        60        70
Brachionus MedPost        KRKHRQVYSRHQTFELEKEYCYSKYLTRKRRVEIAGSIKLTERQVKIWFQNRRMKEKREVGKQSHLIGSIGM Rotifera/Monogononta
Adineta MedPost           RRKHRQVYTRAQTFELEKEYRFSQYLTRKRRSEIAGAVQLSDRQVKIWFQNRRMKEKRENLKFNSAVSTSKQ Rotifera/Bdelloida
Flaccisagitta MedPost     HRERRQTYTRHQTAELEREYVTNRYLTRRRRIEISQSLHLSERQIKIWFQNRRMKEKREKDHVTSPRKNK-- Chaetognatha
Spadella MedPost          HRKRRQTYSRQQTAELEREYVSNRYLTRRRRIEISQHLDLSERQIKIWFQNRRMKERREKDHPATTTTSPRK Chaetognatha
```

Supplementary Figure 3 | Alignment of *Hox* gene homeodomains and 3' flanking regions of paralog group 8 and 9-15 in comparison with the MedPost sequence recovered from *Brachionus manjavacas*. Dashes represent identity with respective *Hox* paraloges from *Capitella teleta* and *Cupiennius salei* shown in the top line of each group. Ecdysozoan genes listed in red, lophotrochozoan genes listed in blue and deuterostome genes listed in green respectively. Residues diagnostic for central class *Hox* genes shared by sequences recovered from *Brachionus majavacas* are highlighted in dark yellow and those representing residues diagnostic for posterior class *Hox* genes are enclosed in green boxes. In the summary residues recovered in rotifers or chaetognaths only are marked in lighter colors respectively.

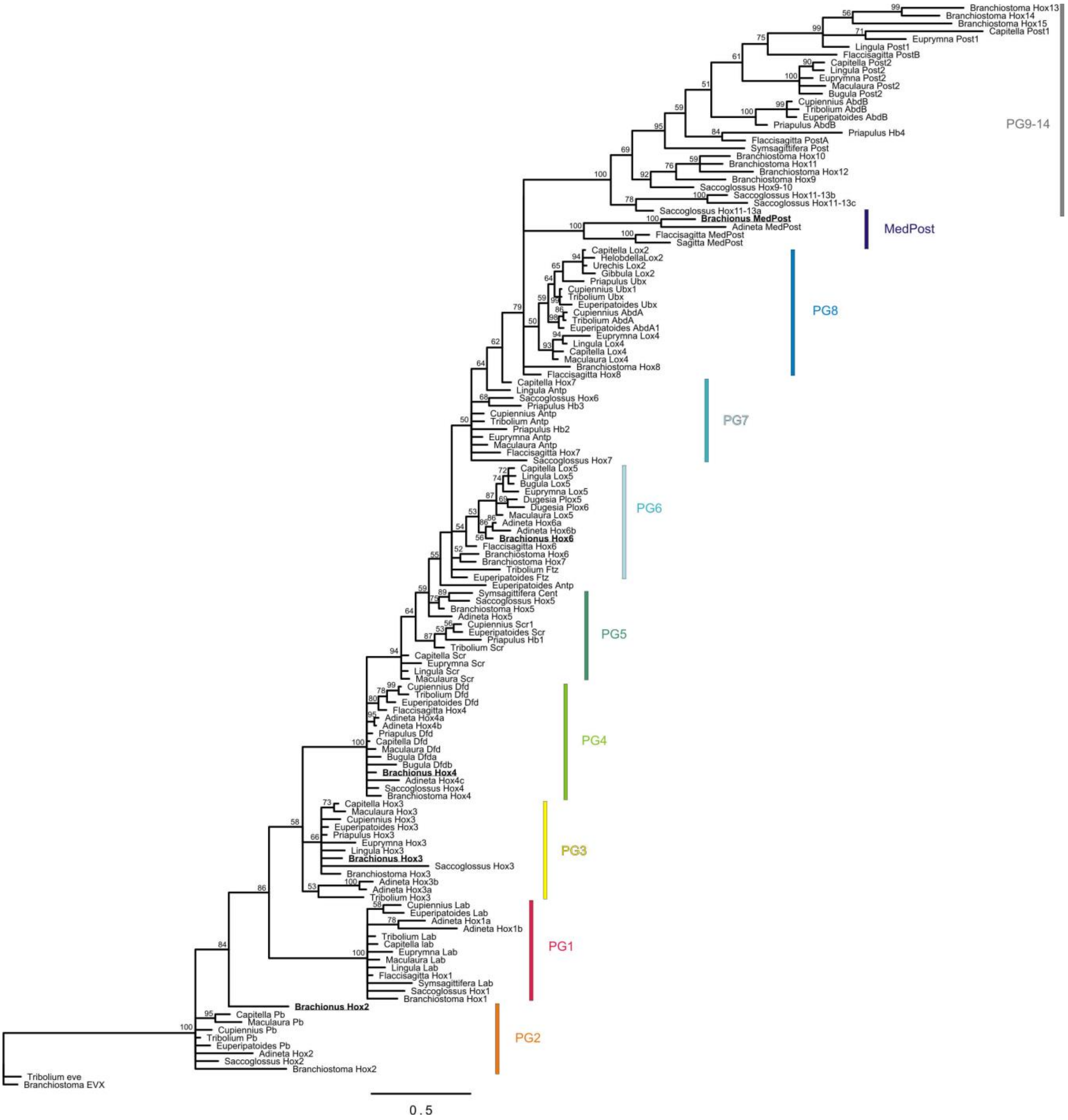

Supplementary Figure 4 | Orthology assignments for *Brachionus plicatilis Hox* genes. Alignments of the 60 amino acid homeodomain and the 12 amino-acids 3’ of the homeodomain of representative bilaterian *Hox* genes were used to conduct a Bayesian phylogenetic analysis based on 400,000 trees from 40,000,000 stationary generations. Bayesian posterior probabilities are shown above the branches. Sequences used for this analysis had been isolated from the following species: *Saccoglossus kowalevskii* (Hemichordata) and *Branchiostoma floridae* (Chordata), *Cupiennius salei* (Chelicerata), *Tribolium castaneum* (Insecta), *Euperipatoides kanangrensis* (Onychophora) and *Priapulus caudatus* (Priapulida), *Capitella teleta* (Annelida/Polychaeta), *Helobdella robusta* (Annelida/Hirudinea), *Urechis unicinctus* (Annelida/Echiurida), *Euprymna scolopes* (Mollusca/Cephalopoda), *Gibbula varia* (Mollusca/Gastropoda), *Maculaura alaskensis* (Nemertea), *Lingula anatina* (Brachiopoda), *Bugula turrita* (Bryozoa) and *Dugesia japonica* (Platyhelminthes), *Symsagittifera roscoffensis* (Xenacoelomorpha), *Flaccisagitta enflata* and *Spadella cephaloptera* (Chaetognatha) and *Brachionus manjavacas* and *Adineta vaga* (monogont and bdelloid rotifers respectively). Sequences from *B. manjavacas* are shown underlined and in bold. *B. manjavacas* possesses orthologs from *Hox* paralogy groups (PG) 2, 3, 4 and 6 and an ortholog of *MedPost*.

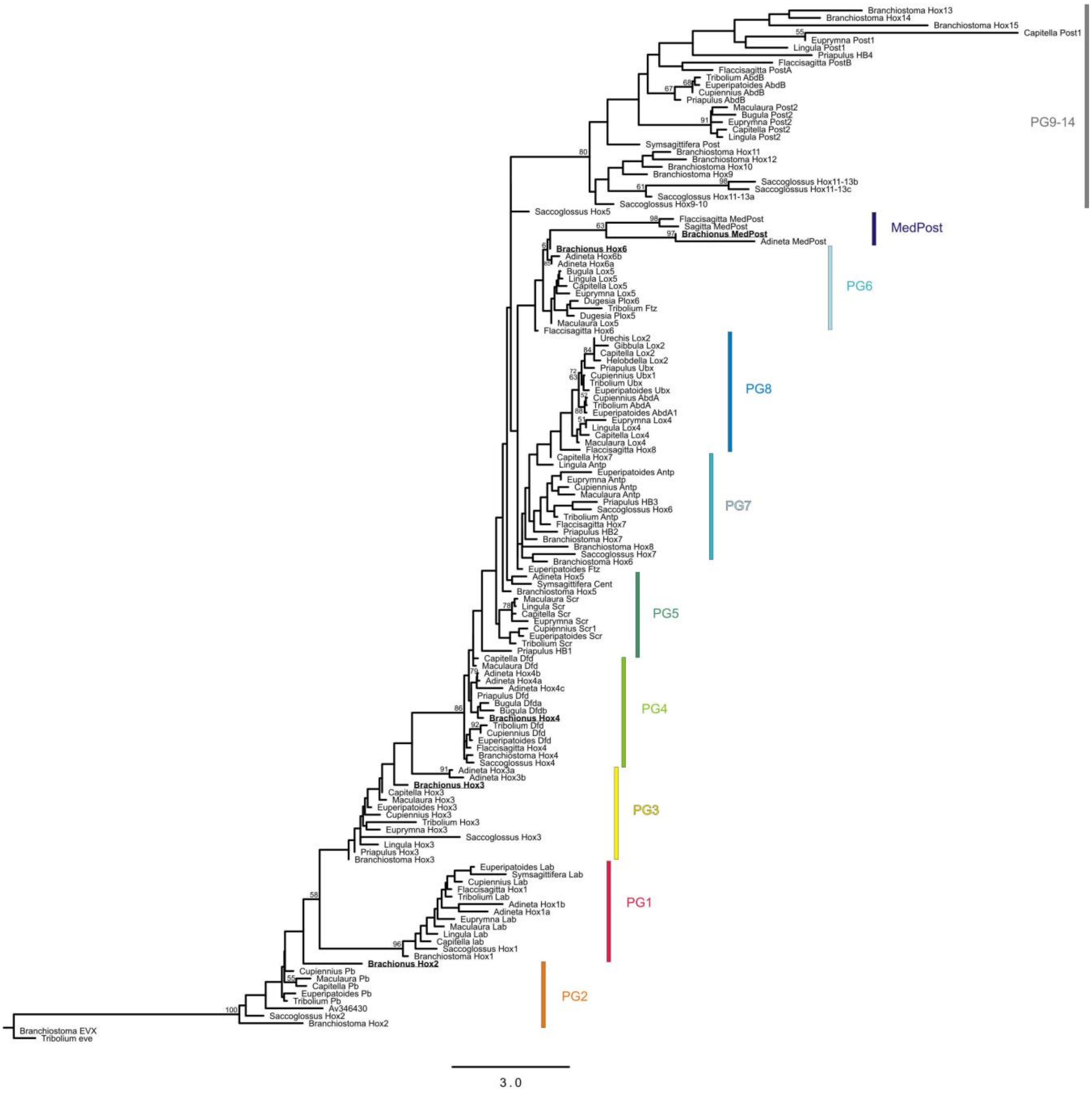

Supplementary Figure 5 | Orthology assignments for *Brachionus manjavacas Hox* genes. Alignments of the 60 amino acid homeodomain and the 12 amino-acids 3’ of the homeodomain of representative bilaterian Hox genes were used to conduct an maximum-likelihood analysis with RAxML-HPC V8.2.9 under the LG+Γ+I model. Bootstrap support values >50 from 1000 iterations are shown above the branches. Sequences used for this analysis had been isolated from the following species: *Saccoglossus kowalevskii* (Hemichordata) and *Branchiostoma floridae* (Chordata), *Cupiennius salei* (Chelicerata), *Tribolium castaneum* (Insecta), *Euperipatoides kanangrensis* (Onychophora) and *Priapulus caudatus* (Priapulida), *Capitella teleta* (Annelida/Polychaeta), *Helobdella robusta* (Annelida/Hirudinea), *Urechis unicinctus* (Annelida/Echiurida), *Euprymna scolopes* (Mollusca/Cephalopoda), *Gibbula varia* (Mollusca/Gastropoda), *Maculaura alaskensis* (Nemertea), *Lingula anatina* (Brachiopoda), *Bugula turrita* (Bryozoa) and *Dugesia japonica* (Platyhelminthes), *Symsagittifera roscoffensis* (Xenacoelomorpha), *Flaccisagitta enflata* and *Spadella cephaloptera* (Chaetognatha) and *Brachionus manjavacas* and *Adineta vaga* (monogonont and bdelloid rotifers respectively). Sequences from *B. manjavacas* are shown underlined and in bold. *B. manjavacas* possesses orthologs from *Hox* paralogy groups 2, 3, 4 and 6 and an ortholog of *MedPost*.

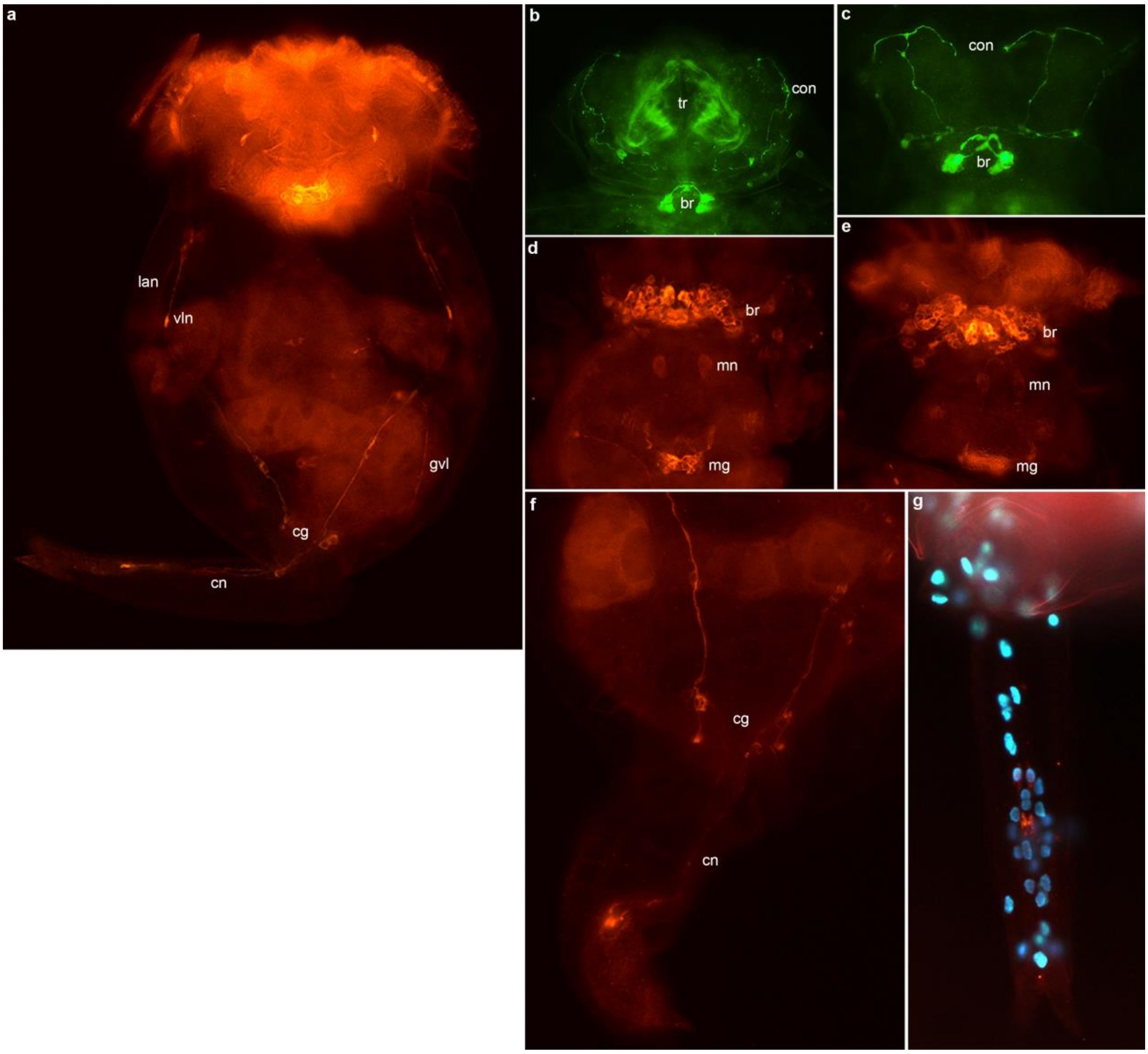

Supplementary Figure 6 | Morphological features of the nervous system of the monogonont rotifer *Brachionus manjavacas* revealed by immunohistochemical staining on adult females. Red staining: labeling of neurons with anti-FRMFamide, green staining: labelling of neurons with anti-5HT. Blue staining: labelling of nuclei with Hoechst 33342. Anterior to the top. Ventral views except for an anteroventral view in **b**. br, brain; cg, caudal ganglion; cn, caudal nerves; con, coronal nerve; gvl, germovitellarial nerve loop; lan, lateral nerves; mg, mastax ganglion; mn, mastax nerve; tr, trophi; ven, ventrolateral nerves.

Supplementary Table 1 | Primers used for isolation of *Hox* gene sequences of *Brachionus manjavacas*.

Primer	***Sequence 5'->3'***	
ELEKEF	GAR YTN GAR AAR GAR TT	universal forward *Hox* primer
WFQNRR	CKN CKR TTY TGR AAC CA	universal reverse *Hox* primer
KLARTAYT	AAG CTT GCC MGN ACN GCN TAY AC	PG1-3 *Hox* primer
AQLVELEKE	GCB CAR YTN GTH GAR YTV GAR AAR G	PG3 primer
RGRQTY	MGN GGN MGN CAR ACN TA	PG4-8 *Hox* primer
BmHox2_RACEfw	TGT GTC GGC CTA GAC GTG TTG AAA TAG CGT CC	*Brachionus Hox2* 3'RACE-primer
BmHox2_RACErev	CAA ATT GGA CGC TAT TTC AAC ACG TCT AGG CCG AC	*Brachionus Hox2* 5'RACE-primer
BmHox3_RACEfw	CTT TAC CGC CTA CTT GTG CAG ACC GCG CCG CAT C	*Brachionus Hox3* 3'RACE-primer
BmHox3_RACErev	TAG TTC GAT GCG GCG CGG TCT GCA CAA GTA GC	*Brachionus Hox3* 5'RACE-primer
BmHox4_RACEfw	CAC CAG GTC CTG GAG CTG GAA AAA GAG TTC CAC	*Brachionus Hox4* 3'RACE-primer
BmHox4_RACErev	GCC TCT CGG ACA AAC TGA GTG TGT GCG CGA TCT C	*Brachionus Hox4* 5'RACE-primer
BmHox6_RACEfw	TGA CTA GAA AGA GGC GAG TTG AAA TTG CCG GAA GC	*Brachionus Hox6* 3'RACE-primer
BmHox6_RACErev	TGC TTC CGG CAA TTT CAA CTC GCC TCT TTC TAG TC	*Brachionus Hox6* 5'RACE-primer
BmMedPost_RACEfw	GCG CCG AAT TGA GAT AGC GCA CTC GTT GGG	*Brachionus MedPost* 3'RACE-primer
BmMedPost_RACErev	ATC TTG ATC TGA CGC TCA GTC AAA CCC AAC GAG TGC	*Brachionus MedPost* 5'RACE-primer

Supplementary Table 2 | Genome accession numbers of *Adineta vaga* genes and Genbank accession numbers of all remaining sequences used in this study.

Species name	Gene	GenBank #. /Genome Acc. #	Species name	Gene	GenBank #. /Genome Acc. #
Adineta vaga	*Adineta Hox1*	Av303240<br>Av041050	*Euprymna scolopes*	*Euprymna Post1*	AAL25811
Adineta vaga	*Adineta Hox2*	Av346430	*Flaccisagitta enflata*	*Flaccisagitta Hox1*	ABS18809
Adineta vaga	*Adineta Hox3*	Av567830<br>Av638170	*Flaccisagitta enflata*	*Flaccisagitta Hox3*	ABS18810
Adineta vaga	*Adineta Hox4*	Av436520<br>Av117920<br>Av439300	*Flaccisagitta enflata*	*Flaccisagitta Hox4*	ABS18811
Adineta vaga	*Adineta Hox5*	Av294680	*Flaccisagitta enflata*	*Flaccisagitta (Hox5)Hox6**	ABS18812
Adineta vaga	*Adineta Hox6*	Av170420<br>Av045650	*Flaccisagitta enflata*	*Flaccisagitta (Hox6)Hox7**	ABS18813
Adineta vaga	*Adineta Medpost*	Av586970	*Flaccisagitta enflata*	*Flaccisagitta Hox8*	ABS18814
Brachionus manjavacas	*Brachionus Hox2*	KT989538	*Flaccisagitta enflata*	*Flaccisagitta MedPost*	ABS18817
Brachionus manjavacas	*Brachionus Hox3*	KT989539	*Flaccisagitta enflata*	*Flaccisagitta postA*	ABS18815
Brachionus manjavacas	*Brachionus Hox4*	KT989540	*Flaccisagitta enflata*	*Flaccisagitta postB*	ABS18816
Brachionus manjavacas	*Brachionus Hox6*	KT989541	*Gibbula varia*	*Gibbula Lox2*	ADJ18238
Brachionus manjavacas	*Brachionus MedPost*	KT989542	*Helobdella robusta*	*Helobdella Lox2*	CAA78665
Branchiostoma lanceolatum	*Branchiostoma Hox1*	ACJ74382	*Lingula anatina*	*Lingula Lab*	AAD45587
Branchiostoma lanceolatum	*Branchiostoma Hox2*	ACJ74381	*Lingula anatina*	*Lingula Hox3*	AAD45588
Branchiostoma lanceolatum	*Branchiostoma Hox3*	ACJ74380	*Lingula anatina*	*Lingula Scr*	AAD45589
Branchiostoma lanceolatum	*Branchiostoma Hox4*	ACJ74383	*Lingula anatina*	*Lingula Antp*	AAD45590
Branchiostoma lanceolatum	*Branchiostoma Hox5*	ACJ74385	*Lingula anatina*	*Lingula Lox5*	AAD45591
Branchiostoma lanceolatum	*Branchiostoma Hox6*	ACJ74384	*Lingula anatina*	*Lingula Lox4*	AAD45593
Branchiostoma lanceolatum	*Branchiostoma Hox7*	ACJ74388	*Lingula anatina*	*Lingula Lox2*	AAD45592
Branchiostoma lanceolatum	*Branchiostoma Hox8*	ACJ74387	*Lingula anatina*	*Lingula Post2*	AAD45595
Branchiostoma lanceolatum	*Branchiostoma Hox9*	ACJ74386	*Lingula anatina*	*Lingula Post1*	AAD45594
Branchiostoma lanceolatum	*Branchiostoma Hox10*	ACJ74389	*Maculaura alaskensis*	*Maculaura Lab*	AKE07580
Branchiostoma floridae	*Branchiostoma Hox11*	AAF81909	*Maculaura alaskensis*	*Maculaura Pb*	AKE07582
Branchiostoma lanceolatum	*Branchiostoma Hox12*	ACJ74391	*Maculaura alaskensis*	*Maculaura Hox3*	AKE07579
Branchiostoma lanceolatum	*Branchiostoma Hox13*	ACJ74390	*Maculaura alaskensis*	*Maculaura Dfd*	AKE07586
Branchiostoma lanceolatum	*Branchiostoma Hox14*	ACJ74393	*Maculaura alaskensis*	*Maculaura Scr*	AKE07583
Branchiostoma lanceolatum	*Branchiostoma Hox15*	ACJ74394	*Maculaura alaskensis*	*Maculaura Lox5*	AKE07585
Bugula turrita	*Bugula Pb*	AAS77225	*Maculaura alaskensis*	*Maculaura Antp*	AKE07577
Bugula turrita	*Bugula Hox3*	AAS77226	*Maculaura alaskensis*	*Maculaura Lox4*	AKE07581
Bugula turrita	*Bugula Dfda*	AAS77227	*Maculaura alaskensis*	*Maculaura Post2*	AKE07584
Bugula turrita	*Bugula Dfdb*	AAS77228	*Priapulus caudatus*	*Priapulus Lab*	AF144884
Bugula turrita	*Bugula Lox5*	AAS77229	*Priapulus caudatus*	*Priapulus Pb*	AF144885
Bugula turrita	*Bugula Post2*	AAS77230	*Priapulus caudatus*	*Priapulus Hox3*	AF144886
Capitella teleta	*Capitella Lab*	EU196537	*Priapulus caudatus*	*Priapulus Dfd*	AF144887
Capitella teleta	*Capitella Pb*	EU196538	*Priapulus caudatus*	*Priapulus HB1*	AF144888
Capitella teleta	*Capitella Hox3*	EU196539	*Priapulus caudatus*	*Priapulus HB2*	AF144889
Capitella teleta	*Capitella Dfd*	EU196540	*Priapulus caudatus*	*Priapulus HB3*	AF144890
Capitella teleta	*Capitella Scr*	EU196541	*Priapulus caudatus*	*Priapulus HB4*	AF144892
Capitella teleta	*Capitella Lox5*	EU196542	*Priapulus caudatus*	*Priapulus HB5*	AF144894
Capitella teleta	*Capitella Antp*	EU196547	*Priapulus caudatus*	*Priapulus Ubx*	AF144891
Capitella teleta	*Capitella Lox4*	EU196543	*Priapulus caudatus*	*Priapulus AbdB*	AF144893
Capitella teleta	*Capitella Lox2*	EU196544	*Saccoglossus kowalevskii*	*Saccoglossus Hox1*	AAP79296
Capitella teleta	*Capitella Post2*	EU196545	*Saccoglossus kowalevskii*	*Saccoglossus Hox2*	ABK00018
Capitella teleta	*Capitella Post1*	EU196546	*Saccoglossus kowalevskii*	*Saccoglossus Hox3*	AAP79286
Cupiennius salei	*Cupiennius Lab*	CAA07497	*Saccoglossus kowalevskii*	*Saccoglossus Hox4*	AAP79297
Cupiennius salei	*Cupiennius Pb*	CAL91855	*Saccoglossus kowalevskii*	*Saccoglossus Hox5*	ABK00019
Cupiennius salei	*Cupiennius Hox3*	CAA06645	*Saccoglossus kowalevskii*	*Saccoglossus Hox6*	ABK00020
Cupiennius salei	*Cupiennius Dfd*	CAA07498	*Saccoglossus kowalevskii*	*Saccoglossus Hox7*	AAP79287
Cupiennius salei	*Cupiennius Scr*	CAL91856	*Saccoglossus kowalevskii*	*Saccoglossus Hox9/10*	ABK00021
Cupiennius salei	*Cupiennius Antp*	CAA07499	*Saccoglossus kowalevskii*	*Saccoglossus Hox11/13a*	ABK00022
Cupiennius salei	*Cupiennius Ubx1*	CAA07500	*Saccoglossus kowalevskii*	*Saccoglossus Hox11/13b*	ABK00023
Cupiennius salei	*Cupiennius AbdA*	CAA07502	*Saccoglossus kowalevskii*	*Saccoglossus Hox11/13c*	AAP79288
Cupiennius salei	*Cupiennius AbdB*	CAB40807	*Spadella cephaloptera*	*Spadella MedPost*	AAO16206
Dugesia japonica	*Dugesia PLOX5*	BAA77405	*Symsagittifera roscoffensis*	*Symsagittifera Lab*	AAN11404
Dugesia japonica	*Dugesia PLOX6*	BAA77406	*Symsagittifera roscoffensis*	*Symsagittifera Central*	AAN11405
Euperipatoides kanangrensis	*Euperipatoides Lab*	CCK73369	*Symsagittifera roscoffensis*	*Symsagittifera post*	AAN11406
Euperipatoides kanangrensis	*Euperipatoides Pb*	CCK73370	*Tribolium castaneum*	*Tribolium Lab*	AAF64148
Euperipatoides kanangrensis	*Euperipatoides Hox3*	CCK73371	*Tribolium castaneum*	*Tribolium Pb*	AAF03888
Euperipatoides kanangrensis	*Euperipatoides Dfd*	CCK73372	*Tribolium castaneum*	*Tribolium Zen*	NP_001036813
Euperipatoides kanangrensis	*Euperipatoides Ftz*	CCK73374	*Tribolium castaneum*	*Tribolium Dfd*	AAK16423
Euperipatoides kanangrensis	*Euperipatoides Scr*	CCK73373	*Tribolium castaneum*	*Tribolium Ftz*	AAK16421
Euperipatoides kanangrensis	*Euperipatoides Antp*	CCK73375	*Tribolium castaneum*	*Tribolium Scr*	AAK16422
Euperipatoides kanangrensis	*Euperipatoides Ubx*	CCK73376	*Tribolium castaneum*	*Tribolium Antp*	EEZ99250
Euperipatoides kanangrensis	*Euperipatoides AbdA*	CCK73377	*Tribolium castaneum*	*Tribolium Ubx*	NP_001034497
Euperipatoides kanangrensis	*Euperipatoides AbdB*	CCK73379	*Tribolium castaneum*	*Tribolium AbdA*	AAB70263
Euprymna scolopes	*Euprymna Lab*	published	*Tribolium castaneum*	*Tribolium AbdB*	AAF36721
Euprymna scolopes	*Euprymna Hox3*	AAL25806	*Urechis unicinctus*	*Urechis*	AAW72807
Euprymna scolopes	*Euprymna Scr*	AAL25807			
Euprymna scolopes	*Euprymna Lox5*	AAL25808			
Euprymna scolopes	*Euprymna Antp*	AAL25809			
Euprymna scolopes	*Euprymna Lox4*	AAL25810			
Euprymna scolopes	*Euprymna Post2*	AAL25812			

* Matus *et al.* (2007)[10] isolated *Flaccisagitta enflata* orthologs of *Hox6* and *Hox7* genes as shown in Fig.3 and Fig.4 of that publication. The sequences however were submitted to Genbank as *Hox5* and *Hox6* respectively even though a *Flaccisagitta Hox5* had not been isolated. All of our phylogenetic analyses identify these genes as the original *Hox6* and *Hox7* orthologs. The assignments shown in this study reflect the original assignments.

Supplementary References

1 Matus, D. Q. *et al.* Broad taxon and gene sampling indicate that chaetognaths are protostomes. *Curr Biol CB* **16**, R575-R576 (2006).

2 Marletaz, F. *et al.* Chaetognath phylogenomics: a protostome with deuterostome-like development. *Curr Biol CB* **16**, R577-R578 (2006).

3 Marletaz, F. *et al.* Chaetognath transcriptome reveals ancestral and unique features among bilaterians. *Genome Biol.* **9**, R94 (2008).

4 Dunn, C. W. *et al.* Broad phylogenomic sampling improves resolution of the animal tree of life. *Nature* **452**, 745-749 (2008).

5 Hejnol, A. *et al.* Assessing the root of bilaterian animals with scalable phylogenomic methods. *Proc. R. Soc. B,* **276**, 4261-4270 (2009).

6 Philippe, H. *et al.* Acoelomorph flatworms are deuterostomes related to *Xenoturbella*. *Nature* **470**, 255-258 (2011).

7 Struck, T. H. *et al.* Platyzoan paraphyly based on phylogenomic data supports a noncoelomate ancestry of Spiralia. *Mol. Biol. Evol.* **31**, 1833-1849 (2014).

8 Laumer, C. E. *et al.* Spiralian phylogeny informs the evolution of microscopic lineages. *Curr. Biol.* **25**, 2000-2006 (2015).

9 Kocot, K. M. *et al.* Phylogenomics of Lophotrochozoa with consideration of systematic error. *Syst. Biol.,* published online ahead of print doi:10.1093/sysbio/syw079 (2016).

10 Matus, D. Q., Halanych, K. M. & Martindale, M. Q. The *Hox* gene complement of a pelagic chaetognath, *Flaccisagitta enflata*. *Integr. Comp. Biol.* **47**, 854-864 (2007).
